# Transcriptional Response of Multidrug-Resistant *Klebsiella pneumoniae* Clinical Isolates to Ciprofloxacin Stress

**DOI:** 10.1155/2021/5570963

**Published:** 2021-11-28

**Authors:** Roman Farooq Alvi, Bilal Aslam, Muhammad Hidayat Rasool, Saima Muzammil, Abu Baker Siddique, Nafeesa Yasmeen, Mohsin Khurshid, Noreen Sarwar, Ahmad Almatroudi, Riaz Hussain, Zulqarnain Baloch

**Affiliations:** ^1^Department of Microbiology, Government College University Faisalabad, Faisalabad, Pakistan; ^2^College of Veterinary Medicine, South China Agricultural University, Guangzhou 610642, China; ^3^Institute of Microbiology, University of Veterinary and Animal Sciences, Lahore, Pakistan; ^4^Department of Medical Laboratories, College of Applied Medical Sciences, Qassim University, Buraydah, Saudi Arabia; ^5^University College of Veterinary and Animal Sciences, The Islamia University of Bahawalpur, Bahawalpur, Pakistan; ^6^Faculty of Life Science and Technology, Kunming University of Science and Technology, Kunming, Yunnan 650500, China

## Abstract

**Background:**

The term “persisters” refers to a small bacterial population that persists during treatment with high antibiotic concentration or dose in the absence of genetic resistance. The present study was designed to investigate the transcriptional response in indigenous *Klebsiella pneumoniae* under the ciprofloxacin stress.

**Methods:**

Isolation and identification of *K. pneumoniae* were carried out through standard microbiological protocols. The characterization of quinolone resistance was performed by estimating the quinolone susceptibility testing, MIC estimation, and detecting the QRDR and PMQR. Transcriptional response of the isolates to ciprofloxacin was determined using qPCR.

**Results:**

Among 34 isolates, 23 (67%) were resistant to ciprofloxacin. Both QRDR (*gyrA* and *gyrB*) and PMQR (*qnrA, qnrB,* and *qnrS*) were detected in the isolates, and all were found resistant to ciprofloxacin. The mRNA levels of both *mutS* and *euTu* under the influence of ciprofloxacin were significantly increased. On ciprofloxacin exposure, the mRNA levels of the DNA damage response element (*mutS*) were raised in a time-dependent fashion. *K. pneumoniae* showed high-level resistance to ciprofloxacin in the presence of mutations in QRDR and PMQR genes.

**Conclusion:**

The transcriptional response revealed the upregulation of DNA repair and protein folding elements (*mutS* and *euTu*) in ciprofloxacin stress and delayed cell division. The ciprofloxacin was found to trigger various stress responses in a time- and concentration-dependent manner.

## 1. Introduction

The term “persisters” refers to a small bacteria population that persists during treatment with high antibiotics concentration or dose in the absence of genetic resistance [1]. There are different host and bacterial potential factors which help the pathogens to evade the host immune system and persist within the host cells. Among them, sublethal antibiotic concentration is one of the potential factors associated with the development of persistent resistant bacterial strains [2]. *Klebsiella pneumoniae* is a Gram-negative bacterium of the Enterobacteriaceae family and associated with a variety of infections in humans and animals. Among Gram-negative bacterial infections, almost one-third of the infections are caused by *Klebsiella pneumoniae* [3]. The emergence of multidrug-resistant (MDR) bacterial strains is a global health concern and very difficult to treat [4]. Sublethal dosage and inadequate regime of antibiotics steer the evolution of resistant superbugs. It has already been established that sublethal concentration of bactericidal *antibiotics* triggers the development of MDR, tolerant, or persistent *Klebsiella pneumoniae* [5].

Generally, bacteria possess a strong defense mechanism against the antibiotics, which triggers the induction of various adaptive responses and reactions at various stages of the cell cycle. Usually, these adaptive responses are known to be the stress response of the bacteria. The particular stress response is incorporated into a flexible adaptive network which is relative to environmental indicators or signals, and it can increase the antibiotic resistance and may maintain the bacterial viability which may ultimately resume the growth of bacteria once again in the optimum environmental conditions [6]. The concentration of antibiotics may be a reason which triggers the global stress response of the bacteria, or it may also generate a target-specific response [7]. Antibiotics themselves may be considered as stressors because they not only interfere with various cellular processes but the evolution of some stress responses in bacteria due to antibiotics has also been reported, which is linked with the emergence of antibiotic resistance [8, 9].

Ciprofloxacin is a broad-spectrum fluoroquinolone antibiotic that hinders the replication of bacterial DNA by impeding DNA gyrase and topoisomerase. Ciprofloxacin is extensively prescribed in the clinical settings of Pakistan to treat various bacterial infections such as typhoid fever and respiratory tract infection [10]. Recently, we have reported the molecular mechanism of resistance against ciprofloxacin in indigenous *Klebsiella pneumoniae* isolates [11]. The present study is designed to investigate the transcriptional response in indigenous *Klebsiella pneumoniae* under the stress of ciprofloxacin, which ultimately is helpful to find out the link between ciprofloxacin stress and its resistance in indigenous *Klebsiella pneumoniae* isolates.

## 2. Methods

### 2.1. Isolation and Identification of *Klebsiella pneumoniae*

The cross-sectional study was performed at the Department of Microbiology, Government College University, Faisalabad, Pakistan. The protocol used in this study was in accordance with the Declaration of Helsinki and was approved by the Ethics Review Committee (ERC) at Government College University, Faisalabad, Pakistan, No: ERC/GCUF/137-18.

All isolates originated from blood cultures were collected from different reference hospitals of Lahore and Faisalabad, the two metropolitan cities of Pakistan. A total of 200 blood samples were collected from patients suspected of bloodstream infections according to standard indications and methods as described elsewhere [12]. A representative selection of the isolates (10%) was sent to the biomedical Research Center, Northwest Minzu University. The isolation of *K. pneumoniae* was carried out from various clinical samples (*N* = 200) by streaking on Hi-Crome *Klebsiella*-Selective Agar Base M 1573 (HIMEDIA®). Standard microbiological procedures were adopted for morphological, cultural, and biochemical profiling of the isolates using the API 20E kit (bioMérieux, France). Additionally, the molecular identification of the isolates was performed through PCR using specific primers against 16S rDNA (Table 1).

### 2.2. Quinolone Susceptibility Testing (QST)

Quinolone susceptibility testing of all the isolates was initially conducted by an E-test (bioMérieux, France) followed by the broth dilution method. Interpretation of results was performed according to the CLSI guideline 2018 [13].

### 2.3. Molecular Characterization of Quinolone Resistance Determinants

Quinolone resistance-determining regions (QRDRs), i.e., *gyrA* and *parC* genes, were identified through PCR, followed by sequencing and mutation detection. Additionally, plasmid-mediated quinolone resistance (PMQR) was also investigated through PCR by detecting various PMQR determinants which include *qnrA, qnrB,* and *qnrS* genes with the help of specific primers (Table 1). All the PCR products were sent to Macrogen Korea for sequencing. Sequence analysis was conducted by software MEGA7. The database named “qnr Numbering and Sequence” which is available online (https://www.lahey.org/qnrstudies) was used for the analysis of PMQR [14]. Lastly, downstream to the BLAST analysis, all the obtained sequences were aligned and submitted to the NCBI (GenBank) for the allotment of accession numbers.

### 2.4. Primer Designing for Transcriptional Studies

The whole-genome sequence of *K. pneumoniae* was obtained from the NCBI for the synthesis of real-time PCR primers. Primers were designed using Primer3web version 4.0.0 (https://primers.ut.ee/). The chemically synthesized primers were evaluated by conducting PCR from the genomic DNA of known *K. pneumonia*.

### 2.5. Bacterial Growth Curve and Ciprofloxacin Persistence Assay

To get the growth patterns of the selected *K. pneumoniae* clinical isolate, the growth curve was built by measuring absorbance as a function of bacterial growth. The isolate was cultivated in liquid nutrient broth (Sigma-Aldrich® USA) under the ciprofloxacin stress, and results were compared with bacteria growing under standard culture conditions.

### 2.6. Synthesis of Complementary DNA

RNA extraction was performed using a Trizol reagent (Invitrogen, Carlsbad, CA, USA) following the manufacturer's directions. Briefly, overnight-grown culture was subjected to centrifuge to get the pellet. The bacterial pellet was resuspended in cold PBS and TRIzol was added @ 0.4 ml/1 × 10^7^ and incubated for 10 min. Afterward, 100 *μ*l chloroform was added, and the cells were incubated for 5 min. Centrifugation of the sample was carried out at 12,000× g (4°C) for 15 min, and the aqueous phase was taken up as RNA [15].

Reverse transcriptase (Revert Aid™MMuLV, Fermentas) according to the manufacturer's instructions was used, and for PCR reaction mixture consisting of 4 *μ*g cellular RNA, 1.0 mM dNTPs, 5 *μ*M random hexamers, and 1 X M-MLuV buffer solution were used. The PCR reaction mixture was placed in a thermal cycler at 65°C for 6 minutes, 4°C for 5 minutes, 25°C for 12 minutes, and 42°C for 3 minutes followed by the addition of MMuLV (200 U/*μ*L, 1 *μ*L) reverse transcriptase enzyme. After the addition of enzymes, the reaction mixture was further incubated at 42°C for 55 minutes. Finally, the reaction was terminated by incubation at 95°C for 7 minutes.

### 2.7. qPCR Application

To determine the mRNA expression levels of different cellular genes, real-time PCR was performed. The reaction mixture (20 *μ*l) consisted of 2X SYBR Green PCR Master Mix (10 *μ*l, Thermo Fisher Scientific, UK), template cDNA (100 ng), and forward (0.2 *μ*l, 20 pmol) and reverse (0.2 *μ*l, 20 pmol) primer. The real-time PCR was performed on a real-time PCR system (Applied Biosystem 7500/7500 Fast, USA). Preset two-step cycling profile consisted of 95°C (initial melting) for 10 minutes, followed by 40 cycles of 95°C (melting) for 15 seconds and 60°C (annealing and amplification) for 1 minute used for the amplification reaction. The obtained data were analyzed using the software (CFX Maestro™) provided by the manufacturer.

## 3. Results

### 3.1. Characterized Quinolone-Resistant *Klebsiella pneumoniae*

The *K. pneumoniae* isolates *n* = 34 (17%) showed the biochemical API 20E (bioMérieux, France) profile including glucose fermentation, indole, H_2_S, and Methyl Red (MR) negative. All isolates were positive to Voges–Proskauer (VP). Additionally, all the positive isolates have hydrolyzed the urea. A PCR product of 1475 bp against 16S rDNA confirmed the molecular identification of *K. pneumoniae* (KY347730.1). Among the positive samples, 23 (67%) isolates were found resistant to ciprofloxacin (Breakpoints; MIC ≥4 *μ*g/ml). Both QRDR (*gyrA* and *gyrB*), as well as PMQR (*qnrA, qnrB,* and *qnrS*), were detected in isolates that were found resistant to ciprofloxacin (Table 2).

In case of mutation, it was observed that gyrA showed a strong mutation (Ser83 ⟶ Ile substitution) responsible for quinolone resistance. It was observed that amino acid present at 83-position is isoleucine (ATC) instead of serine (TCC). Accession numbers that were allotted to the isolates are MF953599 and MF953600.

### 3.2. Transcriptional Response of *Klebsiella pneumoniae* to Ciprofloxacin

**Figure 1:**
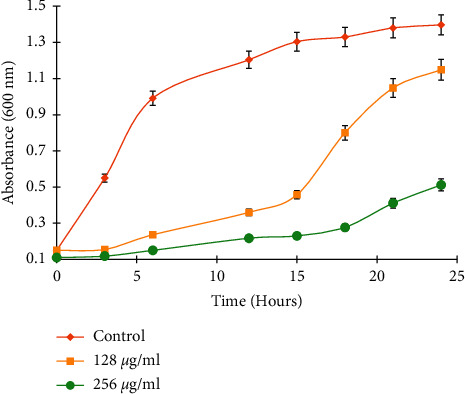
Growth curve of *K. pneumoniae* with ciprofloxacin treatment (conc. 128 *μ*g/ml and 256 *μ*g/ml) and without ciprofloxacin treatment using standard culture conditions. The *X*-axis represents time (hours), whereas the *Y*-axis shows the absorbance (*λ* = 600 nm).

**Figure 2:**
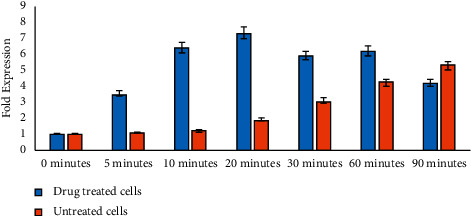
Transcriptomic profiling of *Klebsiella pneumoniae* clinical isolates under ciprofloxacin stress. The graph indicates alterations in the gene expression of euTu, dnaK, mutS, clpB, and lexA in a time-dependent manner. The *X*-axis represents genes, whereas the *Y*-axis displays the fold expression.

In gene expression analysis, we found that the basal expression of DNA repair and protein folding elements under standard growth conditions were increased in the presence of ciprofloxacin (Figure 1). The mRNA levels of both *mutS* (DNA repair gene) and *euTu* (protein synthesis machinery) under the influence of ciprofloxacin were increased to many folds after 5 minutes of exposure and kept on increasing for up to 90 minutes (Figure 2). On *K. pneumoniae* exposure to ciprofloxacin, the mRNA levels of DNA damage response element (*mutS*) increased in a time-dependent fashion.

**Figure 3:**
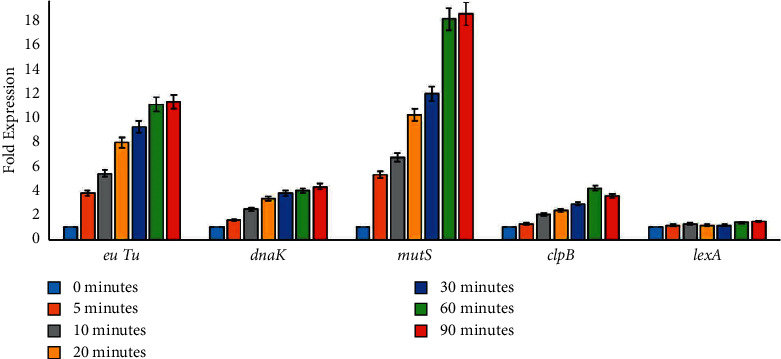
Transcriptomic profiling of *Klebsiella pneumoniae* clinical isolates under ciprofloxacin stress. The graph indicates a change in ftsZ expression in a time-dependent manner. The *X*-axis represents time, whereas the *Y*-axis displays the fold expression.

Moreover, we also found that ciprofloxacin toxicity modulated the expression pattern of chaperones-related genes (*dnaK* and *clpB*) and set off delayed cell divisions (Figure 2). We also observed that the expression of the *dnaK* gene constantly increased up to the initial 90 minutes; however, *clpB* expression declined after the initial 60 minutes. On the contrary, there was no significant change found in *lex*A gene expression (Figure 2). We quantify *ftsZ* gene expression and found that *K. pneumoniae* cell division was delayed due to ciprofloxacin-induced toxicity. Based on real-time PCR results, we observed that ciprofloxacin treatment altered the expression pattern of *ftsZ* gene. In the presence of ciprofloxacin, the expression of *ftsZ* was raised after 90 minutes which supports delayed cell division (Figure 3). Additionally, in current study, the gene expression results were also validated with spectrophotometric analysis, as shown in Figure 2.

## 4. Discussion

The emergence and evolution of multidrug-resistant bacterial strains are a complex issue that is significantly influenced by different factors such as the concentration of antibiotics. Some reports showed that sublethal concentrations of bactericidal antibiotics stimulated the development of MDR bacteria [16]. *K. pneumoniae* is a leading pathogen responsible for severe infections in humans and animals such as pneumonia, soft-tissue infections, and urinary tract infections, and the application of the irrational antibiotic further aggravates its severity. Ciprofloxacin is used extensively in clinical settings of Pakistan. However, *K. pneumoniae* has the ability to resist this antibiotic. The mutation in QRDR, i.e., gyrA and parC genes, is a key player responsible for quinolone resistance. Mutation in QRDR causes structural modifications in enzymes such as DNA gyrase/topoisomerase IV, which decreases the quinolone affinity [17, 18].

*K. pneumoniae* has also shown to produce persister cells by prolonged exposure to antibiotics [5]. According to our best knowledge, no such study has been reported from Pakistan which describes the transcriptional response in *K. pneumoniae* against the irrational antibiotic application in the healthcare settings of Pakistan.

It has already been established that increasing concentration of the antibiotic decreased the ability of *K. pneumoniae* to become a persister, and secondly, the exposure time duration was also a significant factor [5]. The mechanism of persister formation in *K. pneumoniae* is not yet clearly understood, but it is also present in many bacteria. In *E. coli*, quinolone antibiotics regulated the SOS response through *lexA* in a concentration-dependent manner, which is associated with the development of *E. coli* persister cells [19, 20]. This SOS response induction steers the upregulation of various proteins associated with DNA repair which save bacteria from antibiotics and support persister development. On the other hand, DNA damage is the main cause of bacterial death as the concentration of quinolone increased. With extended exposure to quinolone treatment, the persisted bacteria die due to the loss of equilibrium between DNA repair and DNA damage. Though the DNA repair and SOS response are usually conserved in different bacteria, the main phenomenon of SOS response in *K. pneumoniae* still needs to be explored completely. In *E. coli*, the mechanism of persister formation has been elucidated [9]; if this phenomenon is the same in the case of *K. pneumoniae*, then it will be rational to hypothesize that DNA repair possesses a significant role in the formation of *K. pneumoniae* persister associated with ciprofloxacin concentration. Moreover, in the present study, delayed cell division of *K. pneumoniae* was observed in the case of ciprofloxacin treatment, and these findings corroborated the already established stress response elicited in a bacterial cell against antibiotic treatment [8]. Bacteria may exhibit some additional stress responses against antibiotics which may include hyperosmolarity, growth inhibition stress responses, nonoptimal growth temperature, pH downshift, and nutrient starvation [21].

In conclusion, in this study, we found that ciprofloxacin which is the most frequently used antibiotic in clinical settings of Pakistan induces various stress responses in indigenous *K. pneumoniae* isolates in a time- and concentration-depended on manner. Irrational and irregular prescription of this antibiotic may steer the formation of persister *K. pneumoniae* in the community which may ultimately be a serious threat to public health.

## Figures and Tables

**Table 1:** Sequence details of various primers used in the study.

Gene name	Primer sequences
*16S rDNA*	AGAGTTTGATCTGGCTCAG
	AAGGAGGTGWTCCACC

*gyr*A	GGTACACCGTCGCGTACTTT
	TCGATGGAACCGAAGTTACC

*gyr*B	CTCCGTCTCCGTACAGGATGAC
	TGTGATAGCGCAGTTTATCC

*qnrA*	ATTTCTCACGCCAGGATTTG
	GATCGGCAAAGGTTAGGTCA

*qnrB*	GATCGTGAAAGCCAGAAAGG
	ACGATGCCTGGTAGTTGTCC

*qnrS*	ACGACATTCGTCAACTGCAA
	TAAATTGGCACCCTGTAGGC

*lex*A	TCAGTGGGATGTCGATGAAA
	GCCAGACCCTCAATGGTAAA

*eu*-Tu	CAGGTAGGCGTTCCGTACAT
	CCAGCCAGTTCGATGATTTT

*mut*S	GCTGTGGGAATTTGAAATCG
	AGGTACGCTGGGTGTCTTTG

*dna*K	CCAGGACGAAGAAGTTCAGC
	GAAGTAAGCCGGTACGGTGA

*fts*Z	ATGTCAGAAATGGGCTACGC
	ACGGTTTCGAACTCATCCAG

*clp*B	GTACTGCAGCGTCGTACCAA
	GAATGACGTTGCCTTCCTGT

**Table 2:** Minimum inhibitory concentration (MIC) and plasmid-mediated quinolone resistance (PMQR) genes in *Klebsiella pneumoniae* isolates.

Isolate	PMQR gene			MIC of ciprofloxacin (*μ*g/mL)
	*qnr*A	*qnr*B	*qnr*S	
Kp1	−	+	−	64
Kp2	−	+	−	256
Kp3	−	−	+	64
Kp4	−	−	+	32
Kp5	+	−	−	16
Kp6	−	+	+	128
Kp7	−	+	−	64
Kp8	−	−	+	32
Kp9	+	+	−	128
Kp10	−	+	+	256
Kp11	−	+	−	128
Kp12	−	−	+	64
Kp13	−	−	+	64
Kp14	−	−	+	32
Kp15	−	+	−	128
Kp16	−	−	+	32
Kp17	−	+	−	64
Kp18	−	+	−	64
Kp19	−	+	+	128
Kp20	−	+	−	256
Kp21	−	+	−	256
Kp22	−	+	−	64
Kp23	−	−	+	32

## Data Availability

The aggregate data supporting the findings contained within this manuscript will be shared upon request submitted to the corresponding author. Identifying patient data will not be shared.
